# Association between serum sodium and in-hospital mortality among critically ill patients with spontaneous subarachnoid hemorrhage

**DOI:** 10.3389/fneur.2022.1025808

**Published:** 2022-10-31

**Authors:** Dongcai Jin, Shaofeng Jin, Bingyang Liu, Yi Ding, Fen Zhou, Yuhong Jin

**Affiliations:** Department of Critical Care Medicine, Ningbo Medical Center Lihuili Hospital, Ningbo, China

**Keywords:** subarachnoid hemorrhage, sodium, hospital mortality, restricted cubic splines, prognostic factors

## Abstract

**Objective:**

The aim of this study was to retrospectively explore the relationship between serum sodium and in-hospital mortality and related factors in critically ill patients with spontaneous subarachnoid hemorrhage (SAH).

**Methods:**

Data were collected from the Medical Information Mart for Intensive Care IV database. Restricted cubic splines were used to explore the relationship between serum sodium and in-hospital mortality. Receiver operating characteristic analysis was used to calculate the optimal cutoff value of sodium fluctuation, and decision curve analysis was plotted to show the net benefit of different models containing serum sodium.

**Results:**

A total of 295 patients with spontaneous SAH were included in the retrospective analysis. The level of sodium on ICU admission and minimum sodium in the ICU had a statistically significant non-linear relationship with in-hospital mortality (non-linear *P*-value < 0.05, total *P*-value < 0.001). Serum sodium on ICU admission, minimum serum sodium during ICU, and sodium fluctuation were independently associated with in-hospital mortality with odds ratios being 1.23 (95% confidence interval (CI): 1.04–1.45, *P* = 0.013), 1.35 (95% CI: 1.18-1.55, *P* < 0.001), and 1.07 (95% CI: 1.00–1.14, *P* = 0.047), respectively. The optimal cutoff point was 8.5 mmol/L to identify in-hospital death of patients with spontaneous SAH with sodium fluctuation, with an AUC of 0.659 (95% CI 0.573-0.744).

**Conclusion:**

Among patients with spontaneous SAH, we found a J-shaped association between serum sodium on ICU admission and minimum sodium values during ICU with in-hospital mortality. Sodium fluctuation above 8.5 mmol/L was independently associated with in-hospital mortality. These results require being tested in prospective trials.

## Introduction

The most recent Global Burden of Disease (GBD) 2019 stroke burden estimates revealed that stroke remained the second leading cause of death and the third leading cause of death and disability combined in the world in 2019 (1). From 1990 to 2019, the global stroke burden increased substantially, with the bulk of the burden residing in lower-income and lower-middle-income countries (1). This was consistent with relevant studies in China. The stroke burden in China has increased without ceasing over the past 40 years, and in the recent 7 years (from 2013 to 2019), the prevalence of stroke in China has continued to increase (2).

Spontaneous subarachnoid hemorrhage (SAH) is a life-threatening neurological stroke with a high mortality rate of approximately 35% within 30-day (3, 4). In mainland China, the in-hospital death rate was 3.7% for SAH patients (5). Non-traumatic SAH, 85% of cases of which are due to intracranial aneurysm rupture, comprises 3–5% of all stroke types (4, 6, 7). Other non-aneurysmatic rare causes include cerebrovascular malformations and vasculitis, which contains infectious arterial vasculitis and immune vasculitis (8). Many patients with SAH will require admission to the intensive care unit (ICU) for various reasons with higher mortality and disability rates than other ICU patients (7, 9).

Disturbances of sodium balance, namely, dysnatremia, are common and severe complications in critically ill patients (10, 11). Dysnatremia, including hypernatremia and hyponatremia, is the most frequent electrolyte disturbance in SAH, especially in aneurysmal SAH (aSAH), leading to the potential for poor outcomes (12, 13). However, studies enquiring into the association between dysnatremia and neurological outcomes have revealed inconsistent results (14, 15). There are few studies describing the characteristics and potential prognostic impact of sodium variation in critically ill patients with spontaneous SAH in the ICU.

We performed this retrospective cohort study to explore the causal relationship between serum sodium and in-hospital mortality in ICU patients with non-traumatic SAH to better manage electrolytes and improve clinical outcomes ultimately.

## Materials and methods

### Study population

The cohort of critically ill patients with spontaneous SAH' was enrolled from the Medical Information Mart for Intensive Care IV (MIMIC-IV; version 1.0) database, which contains the comprehensive clinical data of 53,130 ICU patients of Beth Israel Deaconess Medical Center between 2008 and 2019. Our Certificate number of permission to use the database is 31101528. It was permitted to dispense with the requirement for informed consent from patients due to the data encryption.

The patients were searched based on the International Classification of Diseases (ICD-10) code. The inclusion criteria identified 420 patients who were diagnosed with SAH. The exclusion criteria were as follows: (1) a definite traumatic etiology (93 patients), (2) ICU readmissions (69 patients), (3) ICU stay of less than 24 h (20 patients), and (4) < 2 sodium measurements available or data missing>5% (12 patients).

### Data source and data collection

Baseline parameters within the first day after ICU admission were collected using Structure Query Language (SQL) with PostgreSQL tools (version 9.6), including demographics (e.g., sex, age, weight, admission type, insurance type, and ethnicity), comorbidities (e.g., myocardial infarction, congestive heart failure, chronic pulmonary disease, and renal disease), assessment scale scores [Glasgow Coma Scale (GCS) and Simplified Acute Physiology Score II (SAPS II)], vital signs (e.g., heart rate, respiratory rate, and blood pressure), the first measured laboratory results at the time of admission to the ICU (e.g., hemoglobin, white blood cell (WBC) counts, platelet counts, serum creatinine (SCr), and blood urine nitrogen (BUN), serum chlorine, serum potassium, and serum sodium), and maximum and minimum serum sodium that was also identified during ICU admission. Sodium fluctuation was calculated by the difference between the sodium highest and lowest levels. In addition, the use of vasopressors, hypertonic saline therapy, and mechanical ventilation were also extracted.

### Statistical analysis

Before analysis, missing data (missing at 0.67–1%) were imputed *via* multiple imputations by chained equations (the mice package for R) to create five imputed datasets.

Continuous variables were analyzed using Student's *t*-test for normally distributed data or the Mann-Whitney *U* test for non-normally distributed data and presented as mean ± standard deviation (SD) or median with interquartile range (IQR). Categorical variables were calculated using the chi-square test or Fisher's exact test and displayed as distribution frequency with percentage.

To further explore the relationship between serum sodium and endpoints, we used restricted cubic splines (RCS), which were adjusted for different kinds of variables or non-adjusted with three knots at the 10th, 50th, and 90th centiles based on logistic regression models using the R package “rms,” if non-linearity was detected. The crude model was not adjusted; the adjusted model 1 was for sex, and age; the adjusted model 2 was for sex, age, Charlson comorbidity index, GCS, SAPS II, white blood cell count, hemoglobin, platelet count, creatinine, serum potassium, and serum chlorine.

Multivariate logistic regression analyses adjusted for potential confounders were performed using a backward/forward stepwise method to investigate the risk factors for hospital mortality. The covariants were selected and determined on statistical significance in the univariable analysis (*P*-value < 0.2) and clinical importance.

Receiver operating characteristic (ROC) analysis using the R package “pROC” was applied to investigate the association of sodium fluctuation with the prognosis of spontaneous SAH. A subgroup analysis was also performed to further analyze the relationship between sodium fluctuation and mortality across different groups of patients, clustered according to their clinical characteristics. The results of the subgroup analysis were presented as forest plots.

To assess the effects of sodium fluctuation on in-hospital mortality, models including sodium fluctuation were analyzed through multivariable logistic regression. In addition to the crude model that only included sodium fluctuation, the models were adjusted for different clinical variables. Moreover, decision curve analysis (DCA) was plotted to show the net benefit and clinical usefulness of different models (16).

A significance was defined as a two-sided *P*-value of < 0.05. Statistical analyses were performed using R version 4.1.2 (R Foundation for Statistical Computing, Vienna, Austria).

## Results

### Baseline characteristics

**Figure 1:**
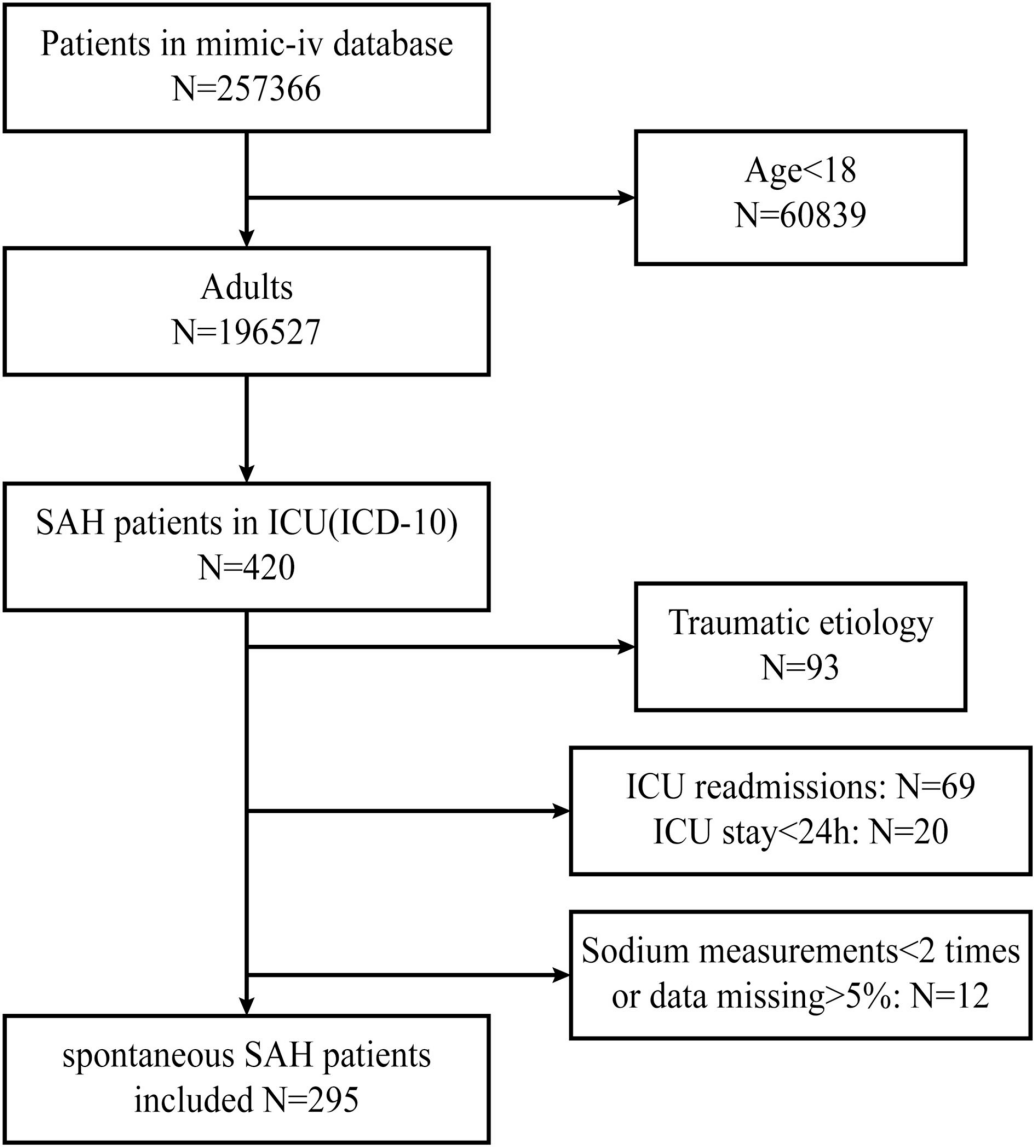
Flowchart screening for eligibility. MIMIC, Medical Information Mart for Intensive Care; SAH, subarachnoid hemorrhage.

The flowchart of enrollment, based on the inclusion and exclusion criteria, is illustrated in Figure 1.

As shown in Table 1, a total of 295 patients with spontaneous SAH were included, 126 of whom were male. The mean age was 60.49 ± 14.73 years. Among these 295 patients, 246 survived and 49 died in the hospital, for an overall in-hospital mortality rate of 16.6%. The non-survivor group was older and had a higher SAPS II score and a lower Charlson comorbidity index compared with the survivor group, with statistically significant differences (*P*-value < 0.05). With regards to laboratory data, WBC count, BUN, creatinine, serum potassium, and serum sodium on ICU admission, minimum sodium, and sodium fluctuation during ICU were statistically significant (*P*-value < 0.05). In addition, the non-survivor group had higher rates of mechanical ventilation, hypertonic saline therapy, and vasoactive agent use, with shorter lengths of stay in the hospital and ICU, all with statistically significant differences (*P*-value < 0.05).

**Table 1:** Baseline characteristics related to in-hospital mortality.

**Variables**	**In-hospital mortality**		***P*-value**
	**Survivors**	**Non-survivors**	
	***N* = 246**	***N* = 49**	
**Baseline characteristics**			
Sex			0.198
Female	145 (58.9)	24 (49)	
Male	101 (41.1)	25 (51)	
Age (year)	59 (51,69)	70 (57,79)	< 0.001
Admission type			0.38
Elective	85 (34.6)	12 (24.5)	
Emergence	130 (52.8)	28 (57.1)	
Emergence surgical	4 (1.6)	1 (2)	
Urgent	27 (11)	8 (16.3)	
Insurance			0.088
Medicaid	13 (5.3)	1 (2)	
Medicare	64 (26)	20 (40.8)	
Other	169 (68.7)	28 (57.1)	
Ethnicity			0.122
White	153 (62.2)	23 (46.9)	
Black/African American	14 (5.7)	3 (6.1)	
Other	79 (32.1)	23 (46.9)	
LOS in hospital (day)	12 (7,20)	4 (2,13)	< 0.001
LOS in ICU (day)	8.6 (4.1,14.7)	3.9 (2.1,11.8)	< 0.001
**Comorbidities**			
Charlson comorbidity index	4 (3,5.8)	5 (4,8)	< 0.001
Myocardial infarction	12 (4.9)	5 (10.2)	0.173
Congestive heart failure	9 (3.7)	7 (14.3)	0.008
Chronic pulmonary disease	33 (13.4)	7 (14.3)	0.871
Renal disease	11 (4.5)	10 (20.4)	< 0.001
**Scoring systems**			
GCS	13 (9,14)	8 (4,15)	0.168
SAPSII	27 (22,33)	37 (30,48)	< 0.001
**Vital signs**			
Heart rate (bpm)	80 (69,90)	79 (68,90)	0.862
SBP (mmHg)	131 (115,145)	132 (118,147)	0.662
DBP (mmHg)	71 (63,79)	74 (64,86)	0.204
MBP (mmHg)	88 (79,98)	90 (82,101)	0.284
Respiratory rate (cpm)	17 (15,20)	18 (16,22)	0.044
**Laboratory tests**			
Hemoglobin (g/L)	12.3 (1.7)	11.9 (1.9)	0.08
White blood cell (10^9^/L)	10.5 (8.4,13.9)	12.4 (10.1,15)	0.006
Platelets (10^9^/L)	209.5 (176.2,250.2)	203 (163,252)	0.492
Creatinine (ng/dL)	0.8 (0.6,0.9)	0.9 (0.7,1.2)	< 0.001
BUN (mmol/L)	13 (10,16)	15 (12,24)	0.001
Cl (mmol/L)	104 (101,106)	105 (100,109)	0.114
K (mmol/L)	3.9 (3.6,4.2)	4.1 (3.7,4.4)	0.039
Na (mmol/L)	139 (137,141)	141 (138,144)	0.001
Minimum Na (mmol/L)	135 (132,137)	138 (134,140)	< 0.001
Na fluctuation (mmol/L)	9 (6,15.8)	16 (9,21)	< 0.001
**Interventions**			
Ventilation	89 (36.2)	44 (89.8)	< 0.001
Vasoactive agent	52 (21.1)	26 (53.1)	< 0.001
Hypertonic saline	58 (23.6)	21 (42.9)	0.005

### Relationship between serum sodium and in-hospital mortality in patients with spontaneous SAH

**Figure 2:**
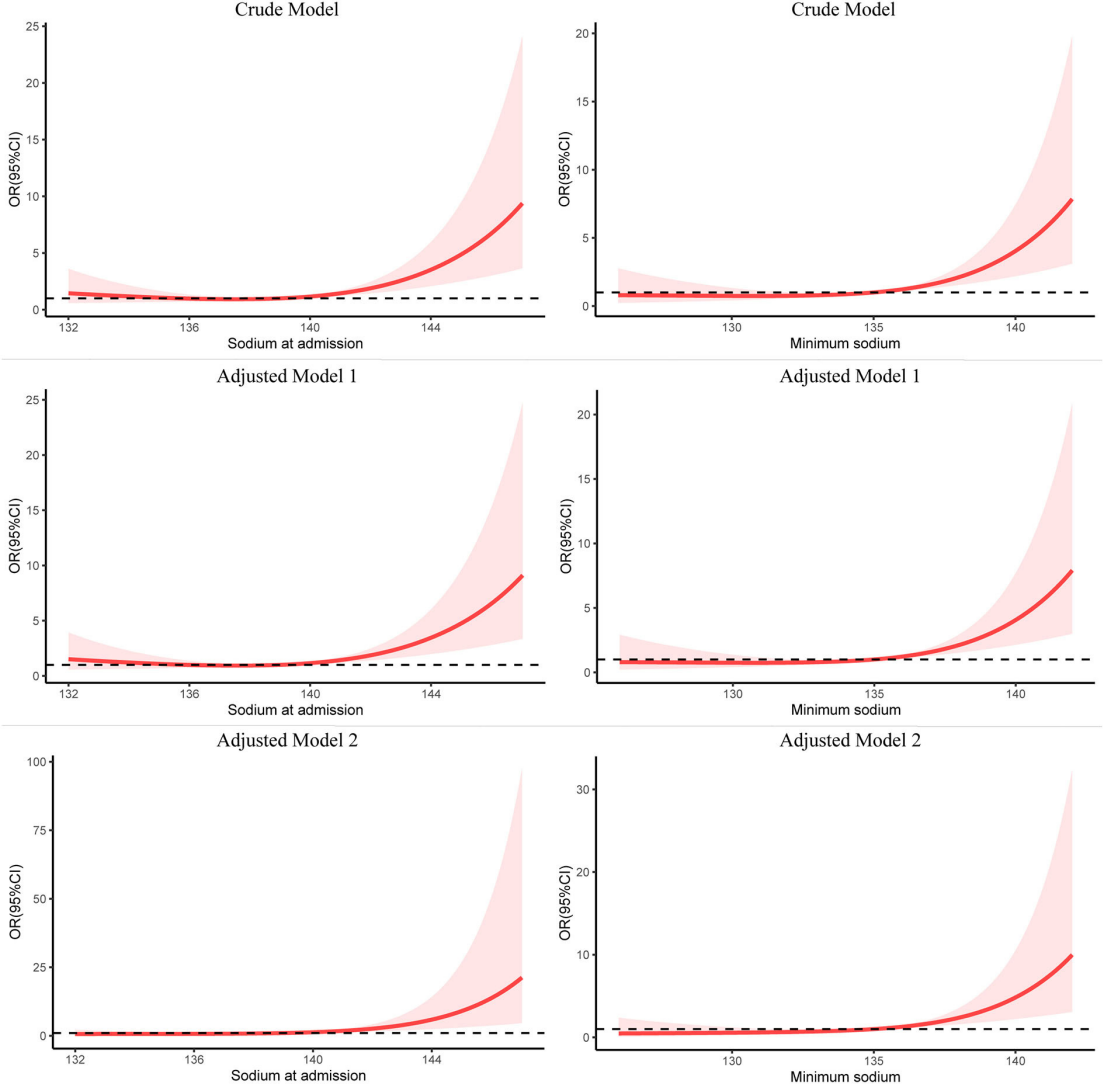
Restricted cubic spline modeling of the relationship between serum sodium and in-hospital mortality in patients with spontaneous SAH. The adjusted model 1 was for sex and age; the adjusted model 2 was for sex, age, Charlson comorbidity index, GCS, SAPS II, white blood cell count, hemoglobin, platelet count, creatinine, K, and Cl. SAH, subarachnoid hemorrhage; RCS, restricted cubic spline; OR, odds ratio.

As shown in Figure 2, RCS analysis indicated that the level of sodium on ICU admission and minimum sodium in the ICU had a statistically significant non-linear relationship with in-hospital mortality in the crude model (non-linear *P*-value < 0.05, total *P*-value < 0.001). The association between sodium on ICU admission and in-hospital mortality was J-shaped, and so was the minimum sodium in the ICU. As the sodium level increased, the risk of in-hospital death increased simultaneously. It could be seen that the growing tendency of mortality became increasingly more pronounced when the level of sodium on ICU admission was above 139 mmol/L, and the minimum sodium in the ICU was higher than 135 mmol/L. After adjusting for the potential confounders in models 1 and 2, RCS analysis still showed the same trend. However, sodium fluctuation in the ICU showed a linear relationship with in-hospital mortality (non-linear *P*-value = 0.53).

### Predicting factors for clinical outcome in patients with spontaneous SAH

Potential risk factors of spontaneous SAH in the univariate and multivariate logistic analysis are presented in Table 2. Serum sodium on ICU admission, minimum serum sodium during ICU, and sodium fluctuation were independently associated with mortality with odds ratios being 1.23 (per mmol/L increase, 95% confidence interval (CI): 1.04–1.45, *P* = 0.013), 1.35 (per mmol/L increase, 95% CI: 1.18–1.55, *P* < 0.001), and 1.07 (per mmol/L increase, 95% CI: 1.00–1.14, *P* = 0.047), respectively. Other variables associated with in-hospital mortality of spontaneous SAH included age, other ethnicities, congestive heart failure, and use of mechanical ventilation.

**Table 2:** Univariate and multivariate logistic analysis between serum sodium level and in-hospital mortality.

**Characteristics**	**Crude OR(95%CI)**	**Crude *P-*value**	**adj.OR (95%CI)**	**adj. *P*-value**
Male	1.50 (0.81–2.77)	0.2		
Age	1.05 (1.02–1.07)	< 0.001	1.08 (1.03–1.13)	0.001
**Admission type**				
Emergence	1.53 (0.74–3.16)	0.26		
Emergence surgical	1.77 (0.18–17.2)	0.62		
Urgent	2.1 (0.78–5.67)	0.14		
**Insurance**				
Medicare	4.06 (0.5–33.01)	0.19		
Other	2.15 (0.27–17.12)	0.47		
**Ethnicity**				
Black/African American	1.43 (0.38–5.35)	0.6	0.37 (0.05–3.02)	0.357
Other	1.94 (1.02–3.67)	0.04	3.16 (1.22–8.18)	0.018
Charlson comorbidity index	1.29 (1.14–1.45)	< 0.001	0.77 (0.57–1.03)	0.083
Myocardial infarction	2.22 (0.74–6.6)	0.15		
Congestive heart failure	4.39 (1.55–12.43)	0.01	24.05 (3.6–160.57)	0.001
Chronic pulmonary disease	1.08 (0.45–2.59)	0.87		
Renal disease	5.48 (2.18–13.76)	< 0.001	6.14 (0.96–39.1)	0.055
GCS	0.87 (0.81–0.94)	< 0.001		
SAPSII	1.08 (1.05–1.11)	< 0.001		
Heart rate	1.01 (0.99–1.02)	0.58		
SBP	1.00 (0.99–1.02)	0.61		
DBP	1.01 (0.99–1.03)	0.23		
MBP	1.01 (0.99–1.02)	0.49		
Respiratory rate	1.06 (1–1.13)	0.05		
Hemoglobin	0.86 (0.72–1.02)	0.08	0.81 (0.62–1.06)	0.12
White blood cell	1.1 (1.03–1.17)	0.01		
Platelets	1.0 (1.0–1.0)	0.96		
Creatinine	1.17 (0.91–1.5)	0.21		
BUN	1.05 (1.02–1.09)	< 0.001		
Cl	1.07 (1.01–1.14)	0.03	0.9 (0.79–1.03)	0.116
K	1.63 (0.95–2.78)	0.07	1.91 (0.84–4.35)	0.122
Na	1.18 (1.09–1.28)	< 0.001	1.23 (1.04–1.45)	0.013
Minimum Na	1.21 (1.11–1.31)	< 0.001	1.35 (1.18–1.55)	< 0.001
Na fluctuation	1.07 (1.03–1.11)	< 0.001	1.07 (1.00–1.14)	0.047
Ventilation	15.52 (5.94–40.58)	< 0.001	23.15 (5.94–90.19)	< 0.001
Vasoactive agent	4.22 (2.23–7.99)	< 0.001		
Hypertonic saline	2.43 (1.28–4.6)	0.01		

### Performance of sodium fluctuation for predicting in-hospital mortality

**Figure 3:**
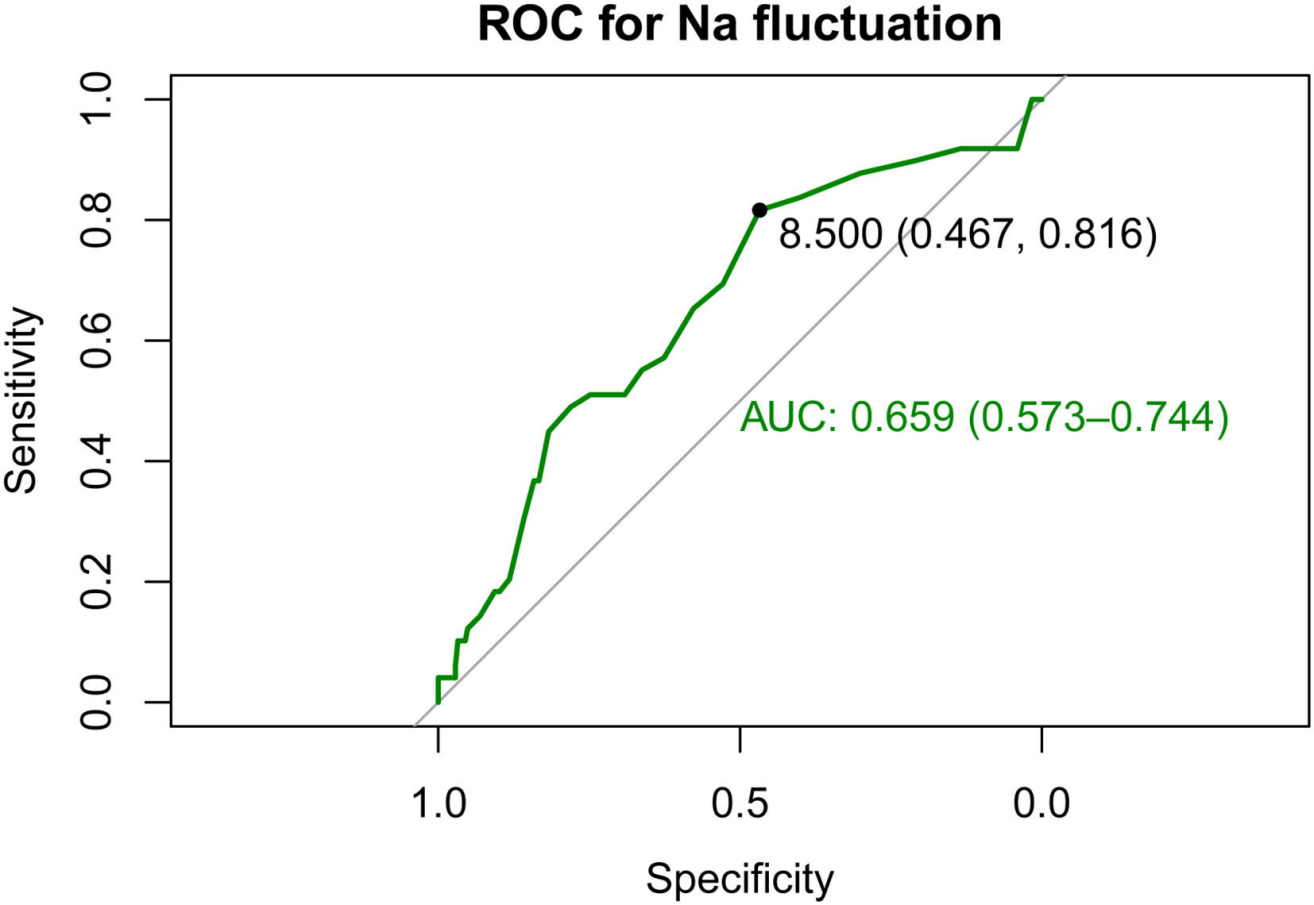
Receiver operating characteristic (ROC) curves show the specificity and sensitivity and the area under the curve (AUC) for predicting in-hospital death with the sodium fluctuation of patients with spontaneous SAH. SAH, subarachnoid hemorrhage.

ROC curve analysis was conducted to calculate the optimal cutoff value of sodium fluctuation and the area under the ROC curve (AUC), as shown in Figure 3. The optimal cutoff point was 8.5 mmol/L to identify in-hospital death of patients with spontaneous SAH with sodium fluctuation. The AUC of sodium fluctuation to discriminate between survivors and non-survivors reached 0.659 (95% CI 0.573–0.744).

When we compared patients with low sodium fluctuation, patients with sodium fluctuation greater than 8.5 mmol/L had a significantly increased in-hospital mortality (23.4 vs. 9.3%, odds ratio, 3.90; 95% CI, 1.89–8.39, *P* < 0.001). After adjusting for other potential confounders, higher sodium fluctuation groups still showed statistical significance. When sodium fluctuation was considered as a continuous variable, logistic regression models showed a positive association with in-hospital mortality.

**Figure 4:**
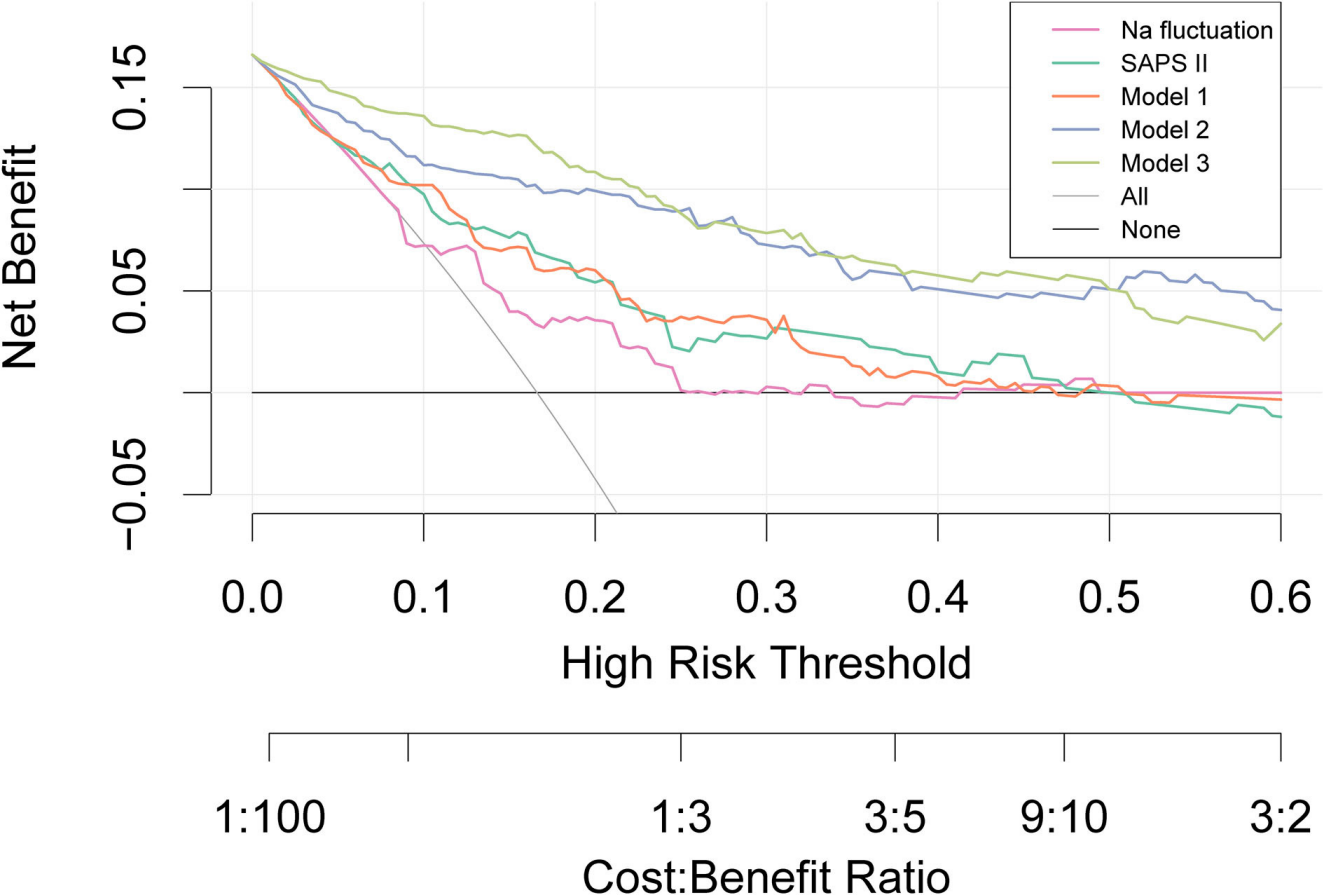
Decision curve analysis. The X-axis indicates the threshold probability for the outcome and the Y-axis indicates the net benefit. Model 1 adjusted for sex, age, and ethnicity. Model 2 adjusted for congestive heart failure, renal disease, respiratory rate, K, Cl, Na, and minimum Na in addition to covariates in Model 1. Model 3 adjusted for ventilation and vasoactive agent in addition to covariates in model 2. SAPS, Simplified Acute Physiology Score.

As shown in Figure 4, DCA showed its clinical usefulness by drawing the net benefit curves of the five models. The X-axis indicates the threshold probability for in-hospital death and the Y-axis indicates the net benefit to stratify the risk of patients. Through DCA, we could discover that model 3 and model 2 always had more net benefits compared with crude sodium fluctuation and other models, and using model 3 added more benefits than model 2 if the threshold probability was less than 20%.

### Subgroup analysis

**Figure 5:**
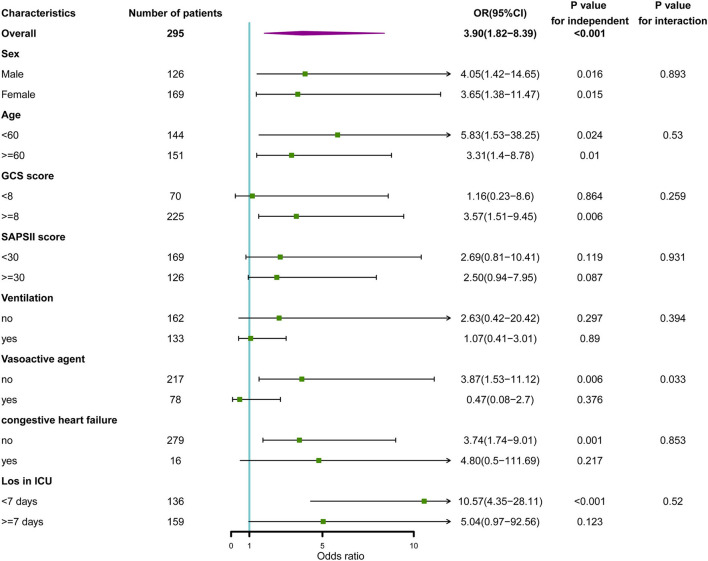
Subgroup analysis of the association between serum sodium and in-hospital mortality. Horizontal lines represent 95% confidence intervals. OR, odds ratio.

In the subgroup analysis between categorical variables of sodium fluctuation and in-hospital death, as shown in Figure 5, we found that only the use of vasoactive agents revealed a differential effect of sodium fluctuation in predicting in-hospital mortality (*P* interaction = 0.033). Patients without vasoactive agent use had a worse prognosis with sodium fluctuation above 8.5 mmol/L.

## Discussion

Our retrospective cohort study demonstrated that in critically ill patients with spontaneous SAH, serum sodium level on ICU admission, minimum sodium in the ICU, and sodium fluctuation in the ICU were independently associated with significantly higher risks of in-hospital mortality. Of these, both serum sodium on ICU admission and minimum sodium values during ICU were in a non-linear relationship with in-hospital mortality, whereas the sodium fluctuation above 8.5 mmol/L represented a higher risk for in-hospital mortality. These associations persisted after adjustment for potential confounders.

Disorders of sodium commonly are encountered in critically ill patients during ICU admission (17). It is well suggested that sodium metabolism is strictly regulated by the kidney through the interaction of many neurohormonal mechanisms, including the renin-angiotoxin-aldosterone system (18). Based on previous studies, hyponatremia in SAH was mostly observed in cerebral salt-wasting syndrome and antidiuretic hormone hypersecretion syndrome, while hypernatremia was also common in critically ill patients with SAH, especially the administration of concentrated NaCl in patients with cerebral edema (19, 20). However, in the current literature, there is no consensus on whether hypernatremia or hyponatremia results in poorer outcomes in patients with SAH.

To the best of our knowledge, it is the first study of its kind to propose a non-linear relationship between serum sodium and in-hospital mortality. Additionally, there was no association between hyponatremia and in-hospital mortality in patients with critically ill spontaneous SAH with serum sodium on ICU admission and minimum sodium in the ICU values below 139 and 135 mmol/L, respectively. This is consistent with previous related studies (21–23). Also, a systematic review concluded that hyponatremia after aSAH was associated with vasospasm and length of hospitalization, but a lack of association with mortality (14). We considered that hyponatremia may be an evolutionary adaptation mechanism in critically ill SAH, as common in other critical illnesses (17). More recently, Chua et al. investigated the relationship between serum sodium levels in the first 14 days post-hemorrhage in patients with aSAH and imaging evidence of vasospasm, neurological deficits, functional outcome, and mortality at hospital discharge and reported that serum sodium values were not significantly different from either vasospasm or in-hospital mortality (24). Similar to our findings, they also discovered that patients who had poorer outcomes including mortality had significantly higher mean sodium levels when looking at the overall mean sodium levels. Our restricted cubic spline analysis showed the increased risk of in-hospital death with the increase of sodium on ICU admission and minimum sodium in the ICU. Similarly, Hoffman et al. reported a significant association between hypernatremia and poor outcomes independent of grade, including adverse cardiac outcomes and death (19). These findings suggested that avoiding hypernatremia may provide neurological, functional, and survival benefits (24).

Several recent studies have focused on the relationship between hospital mortality and sodium fluctuation (10, 25–28). Thongprayoon et al. enrolled 60,944 patients from the Mayo Clinic and found that changes in sodium during the hospital stay were common and significantly associated with hospital and 1-year mortalities after adjusting potential confounders (25). Liang et al. suggested sodium fluctuation as a new single parameter for predicting hospital mortality, and they calculated the optimal cutoffs of 10.5, 15.5, and 16.5 mmol/L for identifying hospital mortality from sodium fluctuation in patients admitted with normonatremia, hyponatremia, and hypernatremia, respectively (27).

Association between variations in serum sodium and aneurysmal subarachnoid hemorrhage (aSAH) has also been reported (23, 24, 29, 30). A retrospective study conducted by Bales and coworkers categorized sodium variability as an absolute maximum change of < 6, 6–12, or >12 mEq/L during intensive care and noted that severe fluctuation in sodium levels was associated with worse neurological outcomes in patients with aSAH (23). Furthermore, Eagles' CONSCIOUS-1 trial demonstrated how the direction of sodium fluctuation affects outcomes, showing that the risk of delayed cerebral ischemia (DCI) after aSAH increased when sodium levels fluctuated in both the positive and negative directions from baseline values on admission. However, multivariate analysis did not suggest that sodium fluctuation was an independent predictor of DCI (29). In our study, we analyzed the relationship between sodium fluctuation during ICU and outcomes in patients with non-traumatic SAH in terms of in-hospital mortality, showing that the optimal cutoff point of sodium fluctuation was 8.5 mmol/L which achieved an AUC value of 0.659. To further verify our findings, both DCA and multivariate analysis revealed the good clinical usefulness and prediction ability of sodium fluctuation. Excessive correction of serum sodium concentration was considered to play a critical role in the pathogenesis of devastating neurological consequences, such as the osmotic demyelination syndrome (ODS), suggesting that the sodium level changes may be of greater importance than the absolute sodium level in critical illness (31). Unfortunately, we have no available data on the occurrence of osmotic demyelination.

In the decision curve analysis, we additionally found that model 2 and model 3 had a similar net benefit and clinical usefulness and were significantly better than the crude model as well as SAPS II. Previous studies have identified that the SAPS II value at admission was a useful and reliable prognosticator in patients with SAH and may provide more information in predicting mortality and poor outcome (32, 33). Similarly, we could draw a conclusion that other than the established predictors such as the Fisher radiological grading scale, Hunt–Hess, and the World Federation of Neurological Surgeons (WFNS), extracerebral organ dysfunction like electrolyte disturbance may play a critical role in predicting outcomes in patients with SAH. Unfortunately, standard grading scales for SAH were not available in the database, and we were unable to include them in this study.

However, this study also has a few limitations. First, as with any retrospective work, this study is limited by the nature of its design. Second, due to the extensive missing data, we did not correct the serum sodium levels measured in patients with hyponatremia to assess the possible impact of blood glucose levels, while there is a consensus that hyperglycemia induces hyponatremia (34). Third, detailed information about hypertonic therapy such as dose and exact timing was not available, which may significantly confound the outcomes for patients with spontaneous SAH with dysnatremia. The lack of these data limited our understanding of the possible conditions that cause serum sodium fluctuation. Fourth, the effects of serum sodium on other patient outcomes, especially neurological outcomes such as the occurrence of DCI, and the modified Rankin Scale (mRS) to assess disability were not included in this study.

## Conclusion

Among patients with spontaneous SAH, we found a J-shaped association between serum sodium on ICU admission and minimum sodium values during ICU with in-hospital mortality, whereas sodium levels above 139 mmol/L on ICU admission and minimum sodium values above 135 mmol/L in ICU were associated with an increased risk of death. Sodium fluctuation above 8.5 mmol/L was independently associated with in-hospital mortality. These results require being tested in prospective trials.

## Data availability statement

The raw data supporting the conclusions of this article will be made available by the authors, without undue reservation.

## Ethics statement

The studies involving human participants were reviewed and approved by Ningbo Medical Center Lihuili Hospital Internal Review Board. Written informed consent for participation was not required for this study in accordance with the national legislation and the institutional requirements.

## Author contributions

DJ and SJ contributed to the conception and study design. DJ, SJ, and BL performed data acquisition. DJ and BL made statistical analyses. DJ drafted the manuscript. SJ, YD, and FZ contributed to the revisions of the manuscript. YJ managed the whole project. The final manuscript was approved by all authors.

## Conflict of interest

The authors declare that the research was conducted in the absence of any commercial or financial relationships that could be construed as a potential conflict of interest.

## Publisher's note

All claims expressed in this article are solely those of the authors and do not necessarily represent those of their affiliated organizations, or those of the publisher, the editors and the reviewers. Any product that may be evaluated in this article, or claim that may be made by its manufacturer, is not guaranteed or endorsed by the publisher.
